# Effects of Open Skill Visuomotor Choice Reaction Time Training on Unanticipated Jump-Landing Stability and Quality: A Randomized Controlled Trial

**DOI:** 10.3389/fnhum.2021.683909

**Published:** 2021-07-29

**Authors:** David Friebe, Tobias Engeroff, Florian Giesche, Daniel Niederer

**Affiliations:** ^1^Division of Preventive and Sports Medicine, Institute of Occupational, Social and Environmental Medicine, Goethe University Frankfurt am Main, Goethe University, Frankfurt, Germany; ^2^Department of Health and Performance, Institute of Occupational, Social and Environmental Medicine, Goethe University Frankfurt am Main, Goethe University, Frankfurt, Germany; ^3^Department of Sports Medicine and Exercise Physiology, Goethe University Frankfurt am Main, Frankfurt, Germany

**Keywords:** open skill exercise, reactive coordination, integrative neuromotor training, injury prevention, anticipation, non-contact injuries, agility

## Abstract

Adapting movements rapidly to unanticipated external stimuli is paramount for athletic performance and to prevent injuries. We investigated the effects of a 4-week open-skill choice-reaction training intervention on unanticipated jump-landings. Physically active adults (*n* = 37; mean age 27, standard deviation 2.7 years, 16 females, 21 males) were randomly allocated to one of two interventions or a control group (CG). Participants in the two intervention groups performed a 4-week visuomotor open skill choice reaction training, one for the upper and one for the lower extremities. Before and after the intervention, two different types of countermovement jumps with landings in split stance position were performed. In the (1) pre-planned condition, we informed the participants regarding the landing position (left or right foot in front position) before the jump. In the (2) unanticipated condition, this information was displayed after take-off (350–600 ms reaction time before landing). Outcomes were landing stability [peak vertical ground reaction force (pGRF) and time to stabilization (TTS)], and landing-related decision-making quality (measured by the number of landing errors). To measure extremity-specific effects, we documented the number of correct hits during the trained drills. A two-factorial (four repeated measures: two conditions, two time factors; three groups) ANCOVA was carried out; conditions = unanticipated versus pre-planned condition, time factors = pre versus post measurement, grouping variable = intervention allocation, co-variates = jumping time and self-report arousal. The training improved performance over the intervention period (upper extremity group: mean of correct choice reaction hits during 5 s drill: +3.0 hits, 95% confidence interval: 2.2–3.9 hits; lower extremity group: +1.6 hits, 0.6–2.6 hits). For pGRF (*F* = 8.4, *p* < 0.001) and landing errors (*F* = 17.1, *p* < 0.001) repeated measures effect occurred. Significantly more landing errors occurred within the unanticipated condition for all groups and measurement days. The effect in pGRF is mostly impacted by between-condition differences in the CG. No between-group or interaction effect was seen for these outcomes: pGRF (*F* = 0.4, *p* = 0.9; *F* = 2.3, *p* = 0.1) landing errors (*F* = 0.5, *p* = 0.6; *F* = 2.3, *p* = 0.1). TTS displayed a repeated measures (*F* = 4.9, *p* < 0.001, worse values under the unanticipated condition, improvement over time) and an interaction effect (*F* = 2.4, *p* = 0.03). Healthy adults can improve their choice reaction task performance by training. As almost no transfer to unanticipated landing successfulness or movement quality occurred, the effect seems to be task-specific. Lower-extremity reactions to unanticipated stimuli may be improved by more specific training regimens.

## Introduction

Adjusting athletic movements (e.g., jump landings) quickly and precisely to unanticipated external visual stimuli is a key demand in interceptive sports (Mache et al., 2013; Almonroeder et al., 2015). Multiple visual stimuli of the environment like positions as well as movements of the opponents, teammates, and equipment must be perceived and processed to initiate a proper motor response (Besier et al., 2001).

Suboptimal decision making and errors in coordination may delay the execution of follow-up actions and can promote injuries (Swanik et al., 2007). Failed landings after a jumping action with a strong external focus, like performing a header in football, represent one of the most common mechanisms of non-contact injuries, like anterior cruciate ligament (ACL) ruptures (Cochrane et al., 2007; Sugimoto et al., 2015). Previous evidence shows that tasks in which an athlete receive a visual cue indicating the side of landing or the direction of a subsequent cutting movement upon landing only briefly before ground contact result in different knee biomechanics, when compared to tasks allowing for sufficient pre-planning. These motor changes during unanticipated landing have been suggested to predispose non-contact ACL injuries (Almonroeder et al., 2015; Hughes and Dai, 2021). For the successful and safe execution of unanticipated athletic movements neuromuscular factors, inhibitory control, and cognitive flexibility (Giesche et al., 2019) have been suggested critical contributors. More detailed, feed-forward and feed-back motor control (Koga et al., 2010; Aerts et al., 2013; DuPrey et al., 2016) as well as cognitive factors, such as processing- and reaction-speed (Herman and Barth, 2016), and visual-spatial memory (Monfort et al., 2019) are named. Improving these abilities may thus lead not only to a better performance but also to a decrease in the injury risk.

To operationalize these landing stability related abilities previous investigations used feed-forward [e.g., peak vertical ground reaction force (pGRF) (Giesche et al., 2019)], as well as feed-back dependent [e.g., time to stabilization (TTS) (DuPrey et al., 2016)] measures. Both TTS and pGRF appear to be directly related to the risk of lower limb injuries (Bakker et al., 2016; DuPrey et al., 2016). In previous trials, decision making performance during unanticipated tasks has been measured by error count (e.g., landing in the wrong position) (Mache et al., 2013; Giesche et al., 2019). First evidence suggests that the ability to successfully react and adapt athletic movements (e.g., landing) to a visual cue under high time constraints rely on cognitive functions, such as working memory and cognitive flexibility (Giesche et al., 2019). The number of decision errors may therefore represent an indirect measure of task-related cognitive function.

As humans act as a single system and not as a compilation of isolated abilities, a combination of motor and cognitive ability training may be the most promising approach to improve unanticipated reactions to external stimuli. As a possibility of such “two for the price of one”-trainings, computerized open skill training devices are often selected (Galpin et al., 2008; Paquette et al., 2017; Wilkerson et al., 2017, 2021; Engeroff et al., 2019, 2020). In contrast to closed skill approaches, which are based on pre-planned movements, athletes in open skill training exercises must adapt their movements to unpredictable external stimuli (Wang et al., 2013).

Device-based open skill training interventions mostly apply choice-reaction drills, executed with either the lower or upper extremities. Lower extremity drills require the athlete to respond to external stimuli while maintaining balance and controlling the body’s center of gravity. This approach seems to result in improved neuromuscular abilities like balance, postural control, and performance in a repeated change of direction task (Galpin et al., 2008; Paquette et al., 2017; Engeroff et al., 2020). On the other hand, upper extremity drills commonly require less balance control as the participants are positioned in a stable bilateral stance in front of the device. This allows them to immediately react to the stimulus without controlling or adapting their posture. Therefore, reaction speed in upper extremity drills seems to rely more directly on the cognitive processing of the stimulus, when compared to the lower extremity drills. This is supported by previous studies indicating that upper extremity drills lead to improved cognitive and visuomotor choice-reaction time (Wilkerson et al., 2017, 2021; Engeroff et al., 2019). As a result of the upper extremity reaction training, Wilkerson et al. (2017) further observed a reduced overall injury incidence.

Although based on the same principles (motor responds to an external stimulus), lower and upper extremity drills may lead to different effects due to different primary demands (Engeroff et al., 2019). Beyond a general need of randomized controlled studies on the effects of such trainings on performance, a direct comparison of upper and lower extremity drills is needed.

Therefore, this investigation compared the effects of an upper and a lower extremity visuomotor open skill choice reaction training on decision-making and landing stability in pre-planned and unanticipated jump landings.

We hypothesized that (1) both trainings lead to improved task specific performance, and that (2) the cognition-dominant upper extremity reaction training is more likely to affect decision making, whereas the neuromotor-dominant lower extremity reaction training is superior in improving landing stability.

## Materials and Methods

### Study Design and Ethical Aspects

This is one part of a three-armed randomized, single-blind controlled experimental design. Other results of this study are already published elsewhere (Engeroff et al., 2019, 2020).

The study was approved by the local ethics commission (reference number: 2016-47). The investigation was conducted in accordance with the Declaration of Helsinki (Version Fortaleza 2013). Before participating in the study, each volunteer signed a written informed consent form.

### Participants

We recruited healthy and physically active (>1 h of exercise per week, assessed by self-report; IPAQ-Short form, Hagströmer et al., 2006) individuals, aged between 18 and 40. Recruiting was undertaken through bulletins, social media, and local sports clubs.

Exclusion criteria were acute or chronic physical or mental illness, as well as injuries and substances abuse. Furthermore, participants were excluded if they had suffered a lower limb injury in the previous 6 months or had undergone surgery in the previous 12 months. Participants were asked to abstain from alcohol and caffeine and to refrain from physical activity for 24 h prior to the pre- and post-examination appointments. Participants were also asked to maintain their regular physical activity habits and regular diet during the study-period.

### Experimental Setup

Participants were randomly allocated to one of the two intervention groups or the control group (CG). The randomization sequence was generated using BiAS 10.0 (BiAS for Windows, Frankfurt), a balanced block-randomization (*n* = 14 per block) was undertaken. The allocation was not concealed.

The two training groups participated in a 4-week open skill visuomotor choice reaction training for the upper or lower extremities. The training sessions were performed in a laboratory of the Institute and were supervised by two sports scientists.

Before and after the 4-week intervention period, all outcomes were assessed. Assessors were blinded to the participants’ group allocation.

### Intervention

Training frequency was: three sessions per week (at least 24 h break between each session) for a total of 12 sessions. Session duration was 20 min. The CG received no intervention.

Before each training session, participants performed a 2-min warm-up, consisting of jumping jacks (= repetitive jumping from neutral stance to a position with legs spread and hands touching overhead).

The training reaction drills were performed on a board (100 × 76 cm) equipped with five sensor pads (top right and left, bottom right, and left, center) connected to a control box that provided a visual stimulus and feedback information via five lights corresponding to the sensor pads (The Quick Board, LCC, Memphis, TN, United States). Galpin et al. (2008) confirmed the reliability of the device (ICC = 0.89).

In one session, three different sets of choice-response tasks were performed, with four trials each. The trials of the first set had a duration of 30 s, those of the second set of 15 s, while the ones of the third set lasted 5 s. Between trials, participants rested in a seated position for 60 s.

For lower extremity training, participants started in an upright position standing on the board. The control box was placed at a distance of 1 m in front of the participants at head level. The feet were positioned to the left and right of the board’s central sensor. No sensor was touched in the starting position. For upper extremity training, the board was placed vertically in front of the participants at head level. The control box was placed between the two upper sensors. After a 5-s countdown, one of the five LEDs representing the sensor areas was activated on the control box. Participants were instructed to tap the respective sensor on the panel with their right or left foot/hand as quickly as possible. The two sensors on the right side had to be touched with the right foot/hand, the sensors on the left side with the left foot/hand. The middle sensor could be touched with either the right or left foot/hand. After a correct contact (placing the correct foot/hand on the indicated sensor pad), the participants had to return to the starting position and another light randomly turned on. The order and selection of the stimulus during all trials was randomized automatically by the device based on a rectangular probability distribution.

**FIGURE 1:**
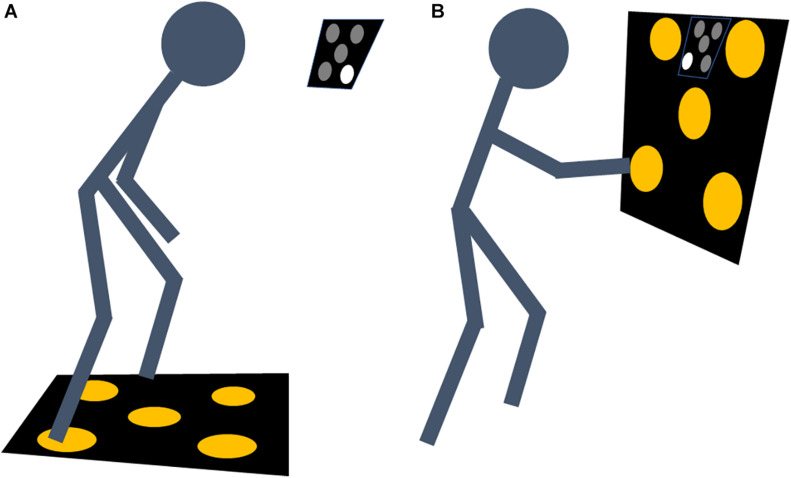
Setup for the open skill visuomotor reaction drill of the lower **(A)** and upper **(B)** extremities.

The goal of each trials was to achieve as many correct contacts as possible. The number of correct contacts was automatically recorded by the device. The setup of the training intervention for the upper and lower extremities is shown in Figure 1.

### Jump Landing Tasks

The participants performed countermovement jumps (CMJ, hands on hips) with pre-planned and unanticipated split-stance landings.

The required landing position (left or right footprint representing the front foot of the split stance) was illustrated on a presentation slide (Microsoft PowerPoint, 2010) displayed on a screen (inch: 17), which was positioned 2 m in front of the participants at chest-height.

In the pre-planned trials, the required landing position was displayed before take-off. For the unanticipated condition, the required landing position was displayed at take-off. For that purpose, a single button USB switch (KKmoon; South Africa) was placed under the jump platform and connected to the laptop. At take-off, the USB switch was activated, leading to a slide change that provided the required landing position (120 ms delay).

In both the pre-planned and unanticipated condition, the participants landed on a capacitive pressure platform. They landed in the required split stance position, aimed to regain a stable stance as quickly as possible, and maintaining this position (hands on hips and fixating a cross at eye-level) for the following 15 s.

To ensure that participants had sufficient time to make a choice-reaction decision (responding to the stimuli) during the jump, we instructed them to jump ∼25–30 cm high (approximately 400–500 ms). The corresponding available reaction time of ∼300–400 ms (flight time minus latency of the automatic stimulus presentation) was comparable to those of previous studies (Giesche et al., 2019). The participants practiced this target jumping height during a familiarization session (pre-planned: *n* = 2, unanticipated *n* = 2) right before the actual measurement. After each jump, we immediately provided them with feedback regarding their achieved flight times to adjust the jumping height, if indicated.

Within the subsequent jumps included in the evaluation, participants performed as many trials as they needed to achieve five successful landings within the pre-planned and unanticipated condition (max. 15 trials for each condition, inter-trial break: was 1 min).

Since wearing shoes, compared to barefoot, can have an impact on postural stability after jump landings, all participants were asked to wear solid sport shoes (Zech et al., 2015).

### Outcomes

During the baseline examination, personal, and anthropometric data as well as the amount of habitual physical activity (IPAQ-Short form; Hagströmer et al., 2006) were assessed. The day of the week and the daytime of pre- and post-measurements were standardized for each participant. Nevertheless, we controlled the participants’ self-reported arousal as a potential confounder. Self-reported arousal was assessed with a visual analog scale from 1 to 10 (Rodenbeck et al., 2001).

Training effects within the open skill visuomotor choice reaction task were operationalized using the mean of the absolute number of correct hits of three 5-, 15-, and 30-s trials.

The landing biomechanics were assessed by a 158 × 60.5 cm capacitive pressure platform (50 Hz, Zebris FDM, Zebris Medical GmbH, Isny, Germany). Gregory and Robertson (2017) reported the Zebris FDM pressure platform to be a valid instrument to assess balance in clinical and research setting (*r* = 0.42–0.66).

Landing stability was operationalized by the peak vertical ground reaction forces [pGRF; (N)] and TTS (sec; Wikstrom et al., 2005). The pGRF represents the maximum value of the recorded vertical ground reaction forces in the *z*-axis. The TTS describes the time required to regain a stable stance after the landings. According to Wikstrom et al. (2005) the stance is defined as stable as soon as the sequential average no longer exceeds the threshold of 0.25 SD of the overall mean ground vertical force. Kaliyamoorthy and Jensen (2009) reported a moderate to high reliability of the TTS. Data on pGRF and TTS were only analyzed for successful trials of the pre-planned and unanticipated landings.

To operationalize the decision-making quality within the unanticipated condition, an investigator documented the number of landing-decision errors (landing on the wrong leg) on an examination form. The jumping height of each trial was calculated via the flight time (assessed by the platform).

### Statistical Analysis

Required participant sample size was estimated using G^∗^Power (Version 3.1.9.2; Germany). Based on the effect size for visuomotor choice reaction training reported in Galpin et al. (2008; Cohen’s *d*: 1.12), an alpha error probability of 0.05 and power of 0.8, we determined a required sample size of 11 participants for each of our three groups. Assuming a dropout rate of 10%, we aimed for a total sample size of 36 participants to be recruited.

All statistical analyses were performed per protocol using SPSS 23 (SPSS Inc., Chicago, IL, United States). Figures and tables were created using Excel 2010 (MS Office, Microsoft Corporation, United States). Results with an alpha-error probability below 5% were considered as statistically significant.

After the range data plausibility check the descriptive analysis was carried out. Mean values as well as standard deviation were calculated. Groups were compared using analysis of variance (ANOVA).

To analyze potential training induced differences between pre- and post-intervention performance between groups within the jump-landing task, a two-factorial (four repeated measures, three groups) ANCOVA was performed for each outcome. The three groups compared included the CG, the lower extremity intervention group as well as the upper extremity intervention group. As repeated measures the two conditions of the jump-landing task (anticipated, unanticipated) as well as the two measurement times (pre and post measurements) were selected. Flight time (difference between pre-planned and unanticipated jumps) and self-reported arousal were set as covariates. Ninety-five percentage confidence intervals of the covariate adjusted means of pre- and post-testing values were used to identify between-condition, time, group, and interaction effects. Confidence intervals not overlapping the mean of the respective comparator are considered significant.

All statistical analysis were conducted after corresponding assumption checks (Shapiro–Wilk test for normality, Levene test for variance homogeneity, linearity, and equal slopes).

## Results

### Descriptive Data

**FIGURE 2:**
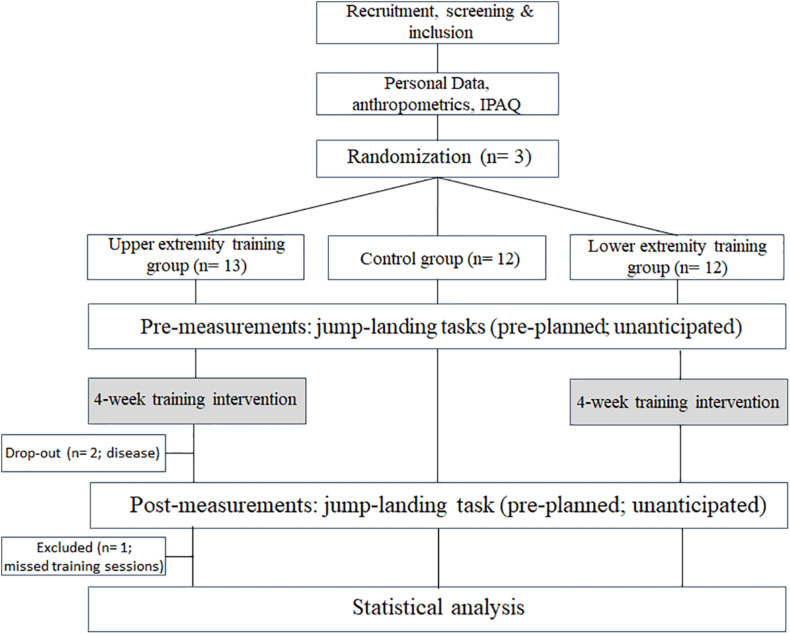
Study and participants flow.

Thirty-seven participants were included into the study. Study and participants flow is displayed in Figure 2. The collective and group-separated participant characteristics are shown in Table 1. Upper extremity intervention group and CG significantly differ in age (*t* = 3.4; *p* = 0.003). No further differences were found between the groups within the anthropometric and health-related data.

**TABLE 1:** Anthropometrics and health related data of the study collective (separated by groups and total sample).

Outcome (mean, SD)	Total sample	Control group	Intervention group upper extremity	Intervention group lower extremity	Between group comparison (*F*-, *p*-value)
Age (years)	27.5, 2.7	29.1, 1.9^#^	26.3, 2.1^#^	27.2, 3.3	5.74, 0.01*
Body height (cm)	173, 10	174, 9.1	172, 12.4	172, 9.1	0.11, 0.89
Body weight (kg)	69.4, 12.1	72.9, 12.0	67.7, 13.7	67.7, 10.6	0.75, 0.48
BMI (kg/m^2^)	23.2, 2.7	24.1, 2.9	22.8, 2.7	22.8, 2.4	0.93, 0.41
Physical activity (MET-hours/week)	46.8, 34.4	57.8, 45.9	45.7, 30.2	35.8, 18.7	1.28, 0.30

*Means and standar**ddeviation** of the BMI, **body-mass-inde**x; cm, centimeter**;** kg, kilogram; m^2^, square meters; MET, metabolic equivalent of task.*

***S**ignificant between-group-differences (*p* ≤ 0.05) are marked with an asterisk.*

*^#^Marks the groups which differ in age.*

**FIGURE 3:**
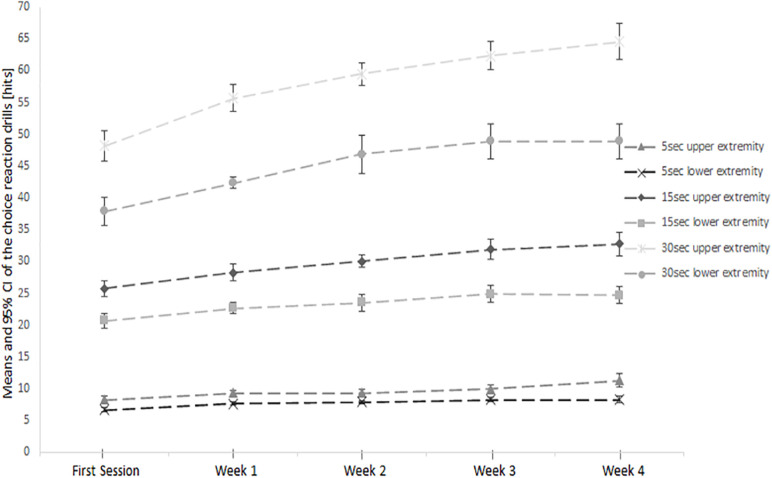
Means and 95% confidence intervals of the absolute number of correct hits in the 15 s open skill visuomotor choice reaction drill during the 4-week training period.

During the intervention, both training groups improved their performance in the 5-s (Upper extremity intervention group: +3.0 ± 1.8 hits; lower extremity intervention group: +1.6 ± 0.843 hits), 15-s (Upper extremity intervention group: +7.0 ± 2.8 hits; lower extremity intervention group: +4.0 ± 2.8 hits), and 30-s (Upper extremity intervention group: +16.3 ± 3.6 hits; lower extremity intervention group: +11.0 ± 5.3 hits) open skill visuomotor choice reaction drill. The weekly progress is displayed in Figure 3.

### Jump Landing Outcomes

Peak ground reaction force showed significant repeated measures (*F* = 6.5; *p* = 0.001) but no group (*F* = 0.4, *p* = 0.9) or interaction (*F* = 2.3, *p* = 0.1) effects.

**FIGURE 4:**
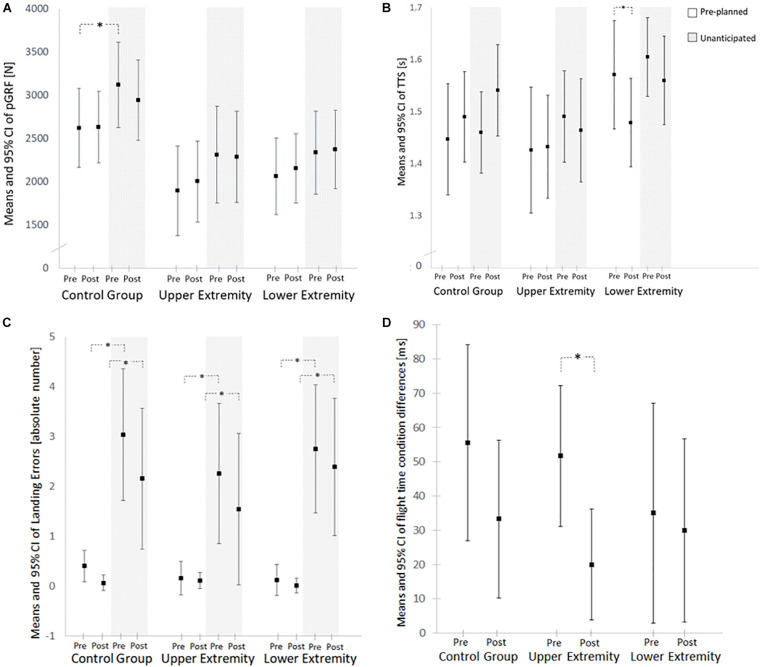
Covariate adjusted means and 95% confidence intervals of the assessed parameters of pre-planned and unanticipated landings. **(A)** Peak ground reaction force (pGRF). **(B)** Time to stabilization (TTS). **(C)** Landing errors. **(D)** Flight time. N, newton; s, seconds; ms, milliseconds. Asterisk marks significant differences (*p* ≤ 0.05).

*Post hoc*, all groups tended to produce higher values under the unanticipated condition with a significantly difference for the CG in the pre-test measurement when compared to the pre-planned condition (CG: +17.4 ± 16.7%; Upper extremity intervention group: +20.4 ± 17.3%; Lower extremity intervention group: +14.8 ± 16.9%) (Figure 4A). Within the post-test measurements, no significant differences between the pre-planned and unanticipated condition appeared (CG: +12.8 ± 21.1%; Upper extremity intervention group: +13.1 ± 18.8%; Lower extremity intervention group: +8.6 ± 13.6%).

Time to stabilization displayed a repeated measures effect (*F* = 4.9, *p* < 0.001).

The lower extremity group showed an improvement over time in the pre-planned condition (CG: +0.6 ± 10.3%; Upper extremity intervention group: −5.2 ± 48.9%; Lower extremity intervention group: −4.7 ± 11.2%), leading to an interaction effect (*F* = 2.4, *p* = 0.03) (Figure 4B).

Between condition differences (unanticipated versus pre-planned landings) in TTS do not appear to be significant in pre- (CG: +1.1 ± 6.0%; Upper extremity intervention group: +4.4 ± 5.9%; Lower extremity intervention group: +1.9 ± 9.4%) and post-test measurements (CG: +4.9 ± 7.8%; Upper extremity intervention group: +1.4 ± 5.2%; Lower extremity intervention group: +4.6 ± 7.7%) (Figure 4B).

Decision making showed a significant repeated measures effect (*F* = 21.8; *p* < 0.001) but no significant between group or interaction effects (*F* = 0.5, *p* = 0.6; *F* = 2.3, *p* = 0.1).

*Post hoc*, all groups displayed a higher absolute number of landing errors in the unanticipated than in the pre-planned condition on pre and post testing day (Figure 4C). Finally, a time × covariate interaction was found for flight time and TTS (*F* = 3.5; *p* = 0.031). In addition, Figure 4 shows a significant decline in the between condition differences (anticipated versus unanticipated) of the flight time following the lower extremity intervention. Absolute values of flight time (pre-planned; unanticipated) were: CG: Pre: 412 ± 60.6 ms; 447 ± 63.4 ms, Post: 414 ± 76.6 ms; 444 ± 64.0 ms; Upper extremity intervention group: Pre: 375 ± 63.3 ms; 418 ± 43.0 ms, Post: 395 ± 52.2 ms; 415 ± 38.0 ms; Lower extremity intervention group: Pre: 382 ± 58.1 ms; 433 ± 67.9 ms, Post: 398 ± 54.9 ms; 418 ± 53.6 ms. Self-reported arousal did not appear to systematically affect any outcome (*p* > 0.05). Values of the self-reported arousal on pre-test measurements were 6.9 ± 1.6 for the CG, 6.5 ± 1.6 for the upper extremity intervention group, and 6.3 ± 1.8 for the lower extremity intervention group. Within in the post testing day participants reported arousal values of 6.4 ± 1.7 in the CG, 7.4 ± 1.1 in the upper extremity intervention group, and 6.4 ± 1.4 in the lower extremity intervention group.

## Discussion

Both intervention groups improved performance within the specific lower or upper extremity open skill visuomotor reaction drill over the 4-week training period. These finding confirm our hypothesis 1.

We found no effect of the lower extremity intervention on the magnitude of the pGRF. However, a shorter TTS under the pre-planned condition after the 4-week lower extremity choice reaction training occurred, which is in line with hypothesis 2. Lacking effects of the upper extremity intervention on decision-making is in contrast to our assumption (hypothesis 2). Nevertheless, a significant reduction in the differences of flight time between the anticipated and unanticipated jump condition occurred after lower extremity training. Since this reduction of flight time did not lead to an increase in landing errors, this might indicate that subjects are able to react faster to a visual stimulus.

The finding of task-specific improvements after visuomotor reaction interventions is consistent with previous studies using the same device or similar devices. After 4-week choice reaction training with upper or lower extremities using the QuickBoard (The Quick Board, LCC, Memphis, TN, United States) Engeroff et al. (2019) found an increased performance within both intervention groups. Furthermore, the lower extremity intervention group improved in the upper extremity drill, but not vice versa. Galpin et al. (2008) and Paquette et al. (2017) confirmed this result regarding the training for lower extremities. Similarly, a visuomotor reaction training with the upper extremities on the Dynavision D2^TM^ System, also led to significant improvements in task-specific performance.

With regard to landing-related outcomes of the present study, conflicting results appear. While the pGRF does not seem to be affected by both interventions, we found a decreased TTS in the pre-planned condition after the lower extremity training. The beneficial adaptation of the postural function is consistent with the results of previous studies. Galpin et al. (2008) found an improvement in pre-planned change of direction speed following 4 weeks of training. In addition, Paquette et al. (2017) found an improved balance and postural control within the star excursion test as well as during a single leg stance after a 6-week lower extremity choice reaction training. Again, the participants showed an enhanced repeated change of direction speed. These findings were confirmed by Engeroff et al. (2020), who found an improvement within the hexagon change of direction test after a 4-week lower extremity choice reaction training. These results indicate that the decrease in the TTS might result from improved feedback and feedforward activation following the lower extremity training. The dynamic neuromuscular demands of the intervention may have led to improved postural control and faster recovery of the body’s center of gravity after athletic movements. In contrast, with our collective and the methodology used, the pGRF does not appear to be sensitive enough to indicate potential effects of the intervention on landing stability regardless condition. Since pGRF is reached within the first 100 ms after ground contact, the improvements in feedback activation and postural control might not be manifested in this outcome (Koga et al., 2010). This result seems to be consistent with the meta-analysis of Lopes et al. (2018). In the context of neuromuscular injury prevention training, they found improvements in landing stability which, however, could not be represented in a reduction of pGRF.

The failed transfer into the unanticipated condition could be due to the high demands on neuromuscular performance and postural control in the lower extremity training intervention. In this training exercise, participants had to return to the starting position after each correct hit before responding to the next stimulus. Therefore, required time for processing and reacting to each stimulus is significantly longer then for the upper extremity drills. The resulting stimuli frequency in such training drills do not appear to reach the critical threshold level for potential neuronal adaptations (Engeroff et al., 2020). Non-specific motor-cognitive drills with complex motor demands and long response times might therefore not be the right choice to address reaction time performance. In contrast, upper extremity training could reach this threshold level but still has no effect on landing stability and landing errors due to missing neuromuscular components in the drills.

The lack of effects of the upper extremity training on the number of landing errors in the unanticipated condition may be due to the complex cognitive and neuromotor demands of the jump-landing task. Here, the participant must perceive an external stimulus, process it, and perform an appropriate lower extremity motor execution. This hypothesis is partly in line with the results of Engeroff et al. (2019), who found improved cognitive reaction time as a result of the same upper extremity reaction training approach, but no carryover to a lower extremity visuomotor reaction drill. The investigations of Wilkerson et al. (2017, 2021) and Williams et al. (2017) confirm the potential for improvements in visuomotor reaction performance following upper extremity choice-reaction training interventions. Williams et al. (2017) also found a significant reduction in core/lower extremity injuries in the following season.

In summary, on the one hand, the lower extremity training appears to increase neuromuscular performance but have only secondary cognitive effects. On the other hand, upper extremity training has a very small motor component but may induce cognitive adaptations. These could possibly not be represented in the number of errors due to lack of adaptations in the speed and quality of motor execution.

The finding of a reduced between condition differences (unanticipated minus pre-planned) in flight time without an increase in landing errors may indicate a transfer effect following the lower extremity training intervention. A reduction of the flight time mean values within the unanticipated condition could be due to the participants feeling more confident in their decision making and/or motor execution, and therefore required a lower jump height (and therefore flight time). In terms of pre-planned jump landings, lower extremity training resulted in participants to choose the solution of a higher jumping height, confident in their improved landing stability.

Beside the treatment effects, we found a distinction in landing stability and decision making between the pre-planned and unanticipated conditions. All groups showed significantly more landing errors within the unanticipated landings in pre- and post-testing measurements. This finding confirms the existing evidence (Giesche et al., 2019). In addition, both outcomes for landing quality showed a trend for worse values in the unanticipated condition with significant differences in pGRF for the CG in the entrance examination. This is consistent with previous studies showing increased pGRF (Meinerz et al., 2015; Yom et al., 2019) during unanticipated athletic movements like jump landings or cutting maneuvers. Insufficient decision-making during athletic movement (Boden et al., 2009), as well as high pGRF (Hewett et al., 2005; Yu et al., 2006; Bakker et al., 2016) and lower postural stabilities (DuPrey et al., 2016; Giesche et al., 2019) upon landing or cutting in unanticipated tasks have been suggested to elevate the risk for non-contact lower limb injuries. This underlines the crucial role of both cognitive and neuromuscular performance for injury prevention open-skilled sports (Koga et al., 2010; Aerts et al., 2013; Herman and Barth, 2016; Giesche et al., 2019; Monfort et al., 2019).

Further studies need to investigate the effect of more sport-specific training paradigms that include more cognitive demanding neuromotor challenging exercises on task performance and injury prevention. Since neither of the two choice-reaction interventions led to improvements in movement quality and decision making within the unanticipated landings on its own, the effect of a combined intervention of motor-cognitive drills with different primary demands (e.g., combination of lower and upper extremity drills) could be examined.

### Limitations

In this study, kinetic data were recorded. These can provide initial insights into the landing biomechanics. However, without kinematic analyses the results cannot be interpreted without doubt regarding the actual loading of the musculoskeletal system. For this purpose, e.g., 3D video recordings or motion capture could be added in future studies to capture joint angles and force moments. A considerable wide range in the flight times occurred (350–600 ms). This may have had an influence on the decision-making capability of each individual. Some participants used a bigger jump height (and therefore a longer flight time) as solution for solving the unanticipated jump-landing task. Because the available response time may affect the cognitive processing demands during the jump, it is difficult attribute potential cognitive improvements to the training intervention. The wide range of the 95% CI of the between-condition differences also demonstrate the heterogeneity of the study population (Figure 4C). Some subjects seem to prefer higher jump heights in the unanticipated condition, while others show similar flight times as in the anticipated condition. The wide range of the 95% CI of the landing errors also confirms the heterogeneity. While some subjects made only a few errors in the unanticipated condition, others seem to have had major problems with the cognitive-motor requirements.

The anticipated and unanticipated jumps are likely to reflect a repeated measure than a second within-subject design. Nevertheless, one may argue that performing a 2 × 2 × 3 ANOVA (instead of our 4 × 3 design) might be appropriate, likewise. This might led to slightly different results in the omnibus tests.

In terms of generalizability, our results give first indications of a transfer of lower extremity training to a sport-related jump-landing task. Nevertheless, this laboratory setting can only reflect the motor-cognitive demands of real game situations to a limited extent. Therefore, future studies should investigate the effect of such open skill reaction training on sport-specific movement patterns and injury risk indicators.

## Conclusion

Our study showed that healthy adults can improve their upper and lower extremity choice-reaction performance by training. Nevertheless, the effect seems to be task specific. While the neuromuscular demanding lower extremity training improves postural stability after pre-planned landings, almost no transfer to decision making and movement quality within the unanticipated condition occurred.

Future studies need to clarify whether and in what context (e.g., rehabilitation, prevention, cognitive/neuromuscular diseases, athletic performance) such exercise paradigms are useful. Furthermore, it should be examined whether visuomotor interventions with more sport-specific stimuli and movements are superior to non-specific choice reaction trainings in terms of improving performance in unanticipated athletic movements.

## Data Availability Statement

The raw data supporting the conclusions of this article will be made available by the authors, without undue reservation.

## Ethics Statement

The studies involving human participants were reviewed and approved by Lokale Ethikkommission des FB05, Goethe-Universität Frankfurt am Main. The patients/participants provided their written informed consent to participate in this study.

## Author Contributions

FG, TE, and DN developed the theory. TE and DF performed the computations. DN and DF performed the statistics and analyses, verified the analytical methods, provided a first manuscript draft, and took the lead in supervising the writing of the manuscript. DF and TE performed the measurements and outcome assessments. TE and DN supervised the findings of this work. All authors conceived of the presented idea, discussed the results, and contributed to the final manuscript.

## Conflict of Interest

The authors declare that the research was conducted in the absence of any commercial or financial relationships that could be construed as a potential conflict of interest.

## Publisher’s Note

All claims expressed in this article are solely those of the authors and do not necessarily represent those of their affiliated organizations, or those of the publisher, the editors and the reviewers. Any product that may be evaluated in this article, or claim that may be made by its manufacturer, is not guaranteed or endorsed by the publisher.
